# Bowel Preparation for Colonoscopy with Sodium Phosphate Solution versus Polyethylene Glycol-Based Lavage: A Multicenter Trial

**DOI:** 10.1155/2008/713521

**Published:** 2008-07-14

**Authors:** S. Schanz, W. Kruis, O. Mickisch, B. Küppers, P. Berg, B. Frick, G. Heiland, D. Hüppe, B. Schenck, H. Horstkotte, A. Winkler

**Affiliations:** ^1^Department of Internal Medicine and Gastroenterology, Evangelic Hospital Kalk, Buchforststrasse 2, 51103 Koeln, Germany; ^2^Gastroenterologische Gemeinschaftspraxis, O7, Nr 14 (Am Wasserturm), 68161 Mannheim, Germany; ^3^Gastroenterologische Gemeinschaftspraxis, Dieburger Strasse 29, 64287 Darmstadt, Germany; ^4^Gastroenterologische Gemeinschaftspraxis, Wiescherstrasse 20, 44623 Herne, Germany; ^5^Gastroenterologische Gemeinschaftspraxis, Schmiedestrasse 18, 30159 Hannover, Germany

## Abstract

*Background*: Adequate bowel preparation is essential for accurate colonoscopy. Both oral sodium phosphate (NaP) and polyethylene glycol-based lavage (PEG-ELS) are used predominantly as bowel cleansing modalities. NaP has gained popularity due to low drinking volume and lower costs. The purpose of this randomized multicenter observer blinded study was to compare three groups of cleansing (NaP, NaP + sennosides, PEG-ELS + sennosides) in reference to tolerability, acceptance, and cleanliness. Patient and Methods: 355 outpatients between 18 and 75 years were randomized into three groups (A, B, C) receiving NaP = A, NaP, and sennosides = B or PEG-ELS and sennosides = C. Gastroenterologists performing colonoscopies were blinded to the type of preparation. All patients documented tolerance and adverse events. Vital signs, premedication, completeness, discomfort, and complications were recorded. A quality score (0–4) of cleanliness was generated. *Results*: The three groups were similar with regard to age, sex, BMI, indication for colonoscopy, and comorbidity. Drinking volumes (L) (A = 4.33 + 1.2, B = 4.56 + 1.18, C = 4.93 + 1.71) were in favor of NaP (*P* = .005). Discomfort from ingested fluid was recorded in A = 39.8% (versus C: *P* = .015), B = 46.6% (versus C: *P* = .147), and C = 54.6%. Differences in tolerability and acceptance between the three groups were statistically not significant. No differences in adverse events and the cleanliness effects occurred in the three groups (*P* = .113). The cleanliness quality scores 0–2 were calculated in A: 77.7%, B: 86.7%, and C: 85.2%. *Conclusions*: These data fail to demonstrate significant differences in tolerability, acceptance, and preparation quality between the three types of bowel preparation for colonoscopy. Cleansing with NaP was not superior to PEG-ELS.

## 1. INTRODUCTION

Colonoscopy is performed as a principal diagnostic tool in most colonic disorders. Adequate bowel preparation is essential for proper visualization of mucosa. Superior cleansing minimizes the risks of missed lesions and repeated procedures, and decreases patients' discomfort and costs of colonoscopy [1, 2].

Peroral polyethylene glycol (PEG) solution has been the cleansing agent most used in recent years. This nondigestible, nonabsorbable osmotically balanced laxative lavage solution does not alter fluid and electrolyte balance [3–6]. Despite the improved tolerance to the two-day standard preparation of clear liquids, laxatives, and enemas, the large fluid intake may lead to nausea and abdominal discomfort. 

Sodium phosphate (NaP), a highly osmotic laxative, has also proven to be an effective and well-tolerated agent [7]. Because of the small quantity needed, it has become one of the preferred agents. Over the past decade, numerous studies have demonstrated that oral NaP is even better tolerated and results in more effective colon cleansing compared with PEG [8–13].

Despite its popularity, NaP generally fails to show better efficacy than PEG-ELS.

In some less comprehensive studies, PEG-ELS preparation even yields better results [14–17].

The purpose of this prospective randomized observer-blinded multicenter study was to compare not only tolerability as the primary aim, but also the efficacy and safety profile of the bowel preparation solely using NaP versus NaP and PEG in combination with sennosides as an adjunctive laxative.

## 2. PATIENTS AND METHODS

A total of 360 patients were recruited during a four-month period, of which 355 consecutive outpatients aged between 18 and 75 years were included in the study, undergoing colonoscopy in 5 endoscopy centres after cleansing preparation. Indications for colonoscopy were history or symptoms of intestinal complaints, weight loss, anaemia, or control of persistent diseases. Patients with an urgent indication for colonoscopy, intolerance to one of the used components, or insufficient compliance were excluded from the study. Patients were randomized into three preparation protocols and analysed for tolerability, efficacy, and safety. They were instructed to remain on a clear liquid diet for 24 hours prior to the procedure. According to the type of the study using well-established routine procedures, for legal reasons no formal ethical approval was necessary. The study was conducted in line with the ethical commitments of the hospital. All patients gave written informed consent. The study was sponsored by Ferring, Germany, without any intellectual or substantial input into the study protocol and analysis of results.

## 3. BOWEL PREPARATION

### 3.1. NaP (group A)

NaP (Fleet Phospho-soda, Ferring, Germany) was administered in two doses of 45 mL each, containing 10.8 g disodium phosphate dodecahydrate and 24.4 g sodium dihydrogen phosphate dihydrate. According to the protocol, the first dose was given in the evening before the examination (19:00 p.m.), and the second in the morning before the procedure (7:00 a.m.). Each dose was diluted in 120 mL of cold water followed by other 240 mL of cold water. The day before colonoscopy the patient was encouraged to drink at least 3 L or more of clear liquids.

### 3.2. NaP + sennosides (group B)

In addition to Group A protocol, sennosides preparation (X-Prep, Mundipharma, Germany) as a supplemental laxative was given the day before the procedure (14:00 p.m.). One dose of 75 mL contains 150 mg of standardized hydroxyanthracene-glycosides (sennoside B). Laxative effects start 5–8 hours after the intake. In addition, the patients had to drink at least a glass (240 mL) of cold water. The day before colonoscopy the patient was encouraged to drink at least 3 L or more of clear liquids within the following 4 hours.

### 3.3. PEG-ELS and sennosides (group C)

Patients receiving PEG-ELS were instructed to remain on clear liquids (3-4 L) after a light meal at 13:00 p.m. the day before the examination. One dose of sennosides (X-Prep) was administered at 14:00 p.m.. Before 7:00 a.m. on the day of the procedure the patients were to drink 4 sachets of PEG-ELS (Klean-Prep) diluted in 1 L of water per dose, containing 68.96 g of PEG 3350 with potassium chloride, sodium chloride, sodium hydrogen carbonate, and sodium sulphate. The intake of PEG-ELS (250 mL each within 10–15 minutes) before colonoscopy was continued until cleanliness of faeces was proven or a maximum volume of 4 L was consumed.

Prior to the examination, patients rated their tolerability of the drinking volume, and the entire preparation received a rating of good, moderate, or poor. Adverse events were recorded according to GCP in detail in the case-report form and carefully monitored by the trial investigator and the sponsor. They were also evaluated (none, mild, moderate, and severe) by each patient. All recorded adverse events were specified and listed in the final report.

Demographic data, comorbidity, and medication of the study population were also documented before allocation to the corresponding preparation regimen according to the random list.

Each colonoscopy was performed by an experienced endoscopist blinded with regard to the preparation protocol. Commencement of the endoscopic procedure was between 11:00 a.m. and 1:00 p.m. The acquisition of information about bowel cleansing methods was not permitted. Premedication, vital signs, and complications during the procedure were documented including reasons for incomplete endoscopy. Discomfort of the ingested fluids was qualified in a verbal rating scale as “no, slight, moderate, or strong discomfort.” The quality of cleanliness and visibility of the colonic mucosa were scored according to a modified five-level rating scale used in prior clinical studies [7–9, 17–19] (see Table 1).

### 3.4. Statistics

Statistical analysis was based on group randomisation to enable allocation of each consecutive patient to the predetermined random list. Computer-generated randomisation lists were applied to each participating centre. Each working day was assigned to one treatment group, resulting in the same preparation protocol for all patients studied at a particular day. Comparison of the three preparation modalities A, B, and C was realized by analysis of variance (ANOVA), *t*-test, U-test, *χ* ^2^ (chi-square) test for testing each regimen pair, Kruskal-Wallis test (K-W test), Mantel-Haenszel *X* ^2^ test for additional pair comparison (*X*_MH_^2^: test of linear relation between columns of cross-tables), analysis of factors, and test of equivalence for responder rates. All tests were conducted two-sided. *P* < .05 was considered to demonstrate statistical significance or equivalence, and respective numbers were given as mean ± standard deviation. The primary objective was to demonstrate a 20% superior tolerability of the treatment arm A. If significant, equivalent efficacy of NaP + sennosides (B) compared with PEG-ELS + sennosides (C) was tested (good and excellent effects) on the basis of a level of equivalence of 15% and a success rate of at least 85% of the standard which was defined hypothetically as PEG-ELS + sennosides. If significant, equivalent efficacy of NaP (A) compared with PEG-ELS + sennosides (C) was tested on the basis of a level of equivalence of 15% and a success rate of at least 85% of the standard. Accordingly, group sizes of at least 107 patients were calculated to test superiority with a power of 1−*β* = 0.80. For data management and statistical analyses, the SAS software was used.

## 4. RESULTS

355 out of 360 patients between 17 and 82 years (median age of 59 years) participated in the study with an indication for a complete colonoscopy. Comorbidity was present in 194 patients (54.6%), mostly with cardiovascular diseases (23.1%) followed by diseases of gastrointestinal tract (16.9%) and endocrine or metabolic abnormalities (13.0%). Medication of concomitant diseases was documented in 174 patients (49%). Characteristics of the patients randomized into the three groups (A, B, and C) are outlined in Table 2.

The three groups were similar with regard to age, BMI, indication for colonoscopy, and comorbidities. Compliance with the cleansing protocol was also comparable between groups A (99.2%), B (97.7%), and C (95.7%). All patients were examined in 5 centres. The distribution of patients to each centre (C) was as follows: C1: *n* = 73, C2: *n* = 69, C3: *n* = 36, C4: *n* = 111, and C5: *n* = 67. The reason for the disproportion of patients in the groups A, B, and C was due to lower numbers of colonoscopies in centres 3 and 4 on the examination days randomized to C.

**Figure 1:**
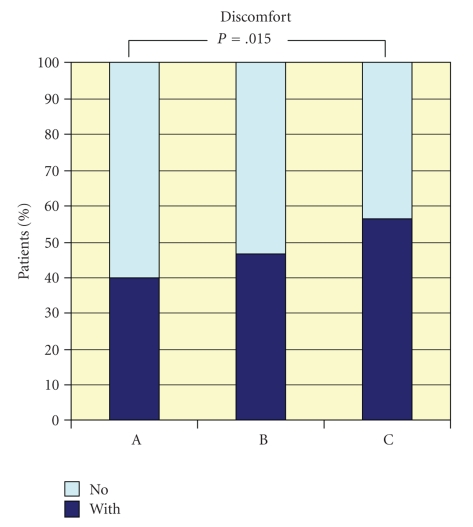
Percentages of patients with (slight to strong) or without discomfort of ingested liquids (*X* ^2^ test: *P* = .015).

The total volume of administered fluids for bowel cleansing in the three groups varied (A: 4.33 L, B: 4.56 L, and C: 4.93 L; ANOVA: *P* = .005, Kruskal-Wallis: *P* = .015). Discomfort according to the verbal rating scale showed a significant difference between Group A and Group C in pair analysis (see Figure 1).

**Figure 2:**
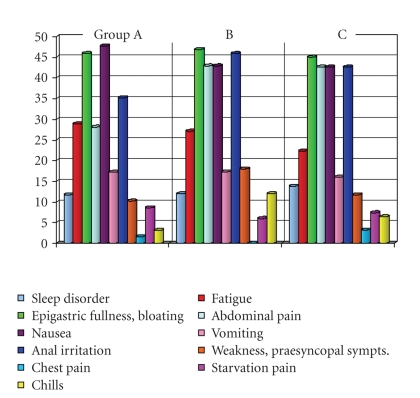
Profile of symptoms during bowel preparation in the three groups (%).

All relevant symptoms during preparation were protocolled (nausea, vomiting, abdominal pain or bloating, anal irritation, fatigue, sleep disorder, hunger, weakness, chest pain, chills). Factor analysis showed heterogeneity between the three groups without relevant differences (see Figure 2). Subgroup analyses showed a higher proportion of patients (>70 years) with nausea and vomiting (nausea: 16 versus 9 patients of >70 years, 41 versus 67 patients of <70 years (K-W test: *P* = .004); vomiting: 9 versus 16 patients of >70 years, 14 versus 94 patients of <70 years (K-W test: *P* = .012)).

**Figure 3:**
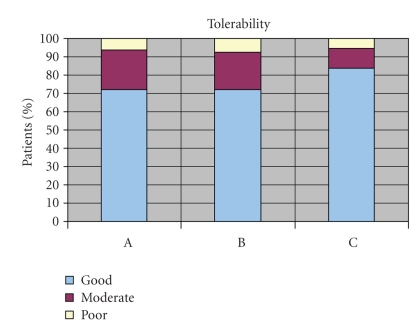
Tolerability of the three preparation modalities received a rating of good, moderate, or poor.

No significant difference in global tolerability of each preparation regimen could be ascertained (see Figure 3).

**Figure 4:**
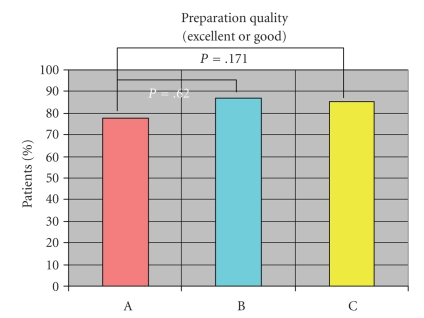
Cleanliness responses to bowel preparation as a percentage between the three groups. Cleansing rating includes excellent and good (*P* = .013).

Complete examination of the colon was possible in 94.9% of the patients. Premedication, vital signs, and pain before and during colonoscopy were registered and were similar in the three groups. The assessment of the cleanliness quality was evaluable in 337 patients in which complete colonoscopy could be achieved. Cleansing efficacy was high in all three groups, and there was no statistical difference. Group C (PEG-ELS + sennosides) tended towards a better response in the analysis of details with regard to all scores (see Figure 4). The rates of good and very good cleanliness quality were 77.7% in Group A, 86.7% in Group B, and 85.2% in Group C (*P* = .135 in *X* ^2^ test) (A versus B: *P* = .062, A versus C: *P* = .171, B versus C: *P* = .755).

Acceptance was high in all groups. Similar proportions of patients were reported to refuse repetition of the same preparation regimen: Group A = 14.8%, Group B = 18.5%, and Group C = 17% (*X* ^2^ test: *P* = .737). If there is alternative preparation offered for colonoscopy, patients would prefer another protocol in A: 30.2%, B: 30%, and C: 37.2% (*X* ^2^ test: *P* = .445).

## 5. DISCUSSION

Colonoscopy is an important tool for the diagnosis and follow-up of colonic disorders and the prevention of neoplasms. There is an ongoing search for the ideal cleansing preparation, aiming at better patient compliance, shorter colonic preparation time, and better cleansing effects. Consumption of a large amount of fluid is frequently associated with poor patient tolerance. It might therefore be expected that a decrease in volume could lead to a better acceptability.

In the present study, bowel cleansing preparation with NaP with or without sennosides was compared to a PEG electrolyte solution with sodium sulphate (PEG-ELS) in combination with sennosides in terms of tolerability, patient acceptance, and bowel cleanliness. The investigation included a considerable number of patients. The differences in the sizes of the three treatment arms are due to the randomisation procedure, and may not very likely influence outcomes in the view of very similar baseline characteristics.

Our results of the present study indicate that all three regimens showed a good tolerability with a trend towards PEG-ELS, but this difference did not reach a level of statistical significance. As demonstrated in other studies, NaP was not better tolerated although the ingested volume of liquids was significantly lower (medium: 14%) in the NaP group. Subgroup analysis in consideration of age however showed significantly more side effects in patients over 70 years like nausea, vomiting, and abdominal pain in Group B (NaP + sennosides). Dizziness and presyncope as symptoms caused by volume depletion and hyperphosphatemia were not predominant in the NaP regimens, which is concordant with the literature [20]. Vomiting and abdominal discomfort reported as frequent side effects of PEG-ELS with a better tolerance of NaP [21] were not increased in the PEG-ELS group.

Acceptance of the three preparation protocols was similar. Less than 20% of each group would not like to repeat the same regimen for bowel cleansing. Also similar proportions of patients would prefer another cleansing solution for the next colonoscopy with an insignificant trend towards NaP protocols. Considerably better results in acceptance were reported in literature when NaP was compared with PEG solutions [8–10, 21–23].

Regarding the cleanliness effects, all three preparation protocols were highly effective with a very good or good response between 77.7% and 86.7%. Overall cleanliness of the colon was assessed without specifying the cleansing effects in the different bowel segments. According to a worst-case analysis, solid faeces in any of the segments was judged as cleanliness score 4 and transparent fluid in all segments as good (score 1). In contrast, Ell et al. [27] analysed the effects of bowel preparation of different segments, which gives some additional information. Because of the high number of patients, we chose a less complex protocol, also omitting systematic videotaping.

Although there was a trend towards better cleanliness with PEG-ELS plus sennosides, the differences showed no statistical significance. Similar responses were reported by others [18, 22–26].

It is widely established to perform bowel cleansing with PEG-ELS within 2 consecutive days. Because of adding sennosides to the preparation protocol taken the day before the examination, there was no distribution of the amount of PEG-ELS on two days with an overnight pause. Hence, in our protocol the whole PEG-ELS dose was taken over at least 4–6 hours on the day of the examination under strict control of the unblinded endoscopy assistant nurses of each centre. Then colonoscopy was commenced.

In a recent study by Ell et al. [27], the PEG-ELS (with sodium sulphate) treatment group demonstrated the optimal cleansing quality in all colon segments compared to PEG-ELS (without sodium sulphate) and NaP. No details were given on the amount of liquids taken together with each preparation regimen, which in our opinion plays an important role in the NaP cleansing. Furthermore, most of the examined subjects were inpatients (86%)—a treatment group with generally more severe illnesses or at least more severe comorbidity which may affect bowel preparation. Therefore, it cannot be compared with our treatment group made up exclusively of outpatients. PEG-ELS doses were administered in two portions on the day before and the day of the examination, which might be an advantage referring to cleanliness of proximal colonic segments [19].

Despite the administration in a two-step procedure with an overnight pause, NaP showed no better results than PEG-ELS plus sennosides as in the study of Frommer [10]. Colonoscopy was performed at the earliest 3-4 hours after the second dose of NaP.

The combination of NaP with sennosides, a commonly used laxative to optimize bowel preparation for diagnostic procedures [28], produces a slightly better cleanliness response than NaP alone. But this minor synergistic effect does not reach statistical significance. On the other hand, there was a tendency towards more side effects, as mentioned above.

A meta-analysis of the trials comparing NaP with PEG solutions concluded that oral NaP was better tolerated by patients and was at least as efficacious [29].

But heterogeneous groups of patients, outpatients, and inpatients and different protocols were mixed up, which limits the conclusion.

Each preparation protocol was carried out according to the dosing instructions recommended for afternoon colonoscopies. Being consistent with the recommendation of the ASGE, patients with severe renal insufficiency or unstable cardiac diseases were excluded from the study [30].

## 6. CONCLUSION

In conclusion, tolerability, acceptance, and the effectiveness of the bowel cleansing regimens for elective colonoscopy with two-dose, low-volume NaP and 4 L PEG-ELS plus sennosides were comparable. Sennosides as adjunctive laxative have only minor synergistic effects when added to NaP, and cannot be recommended for routine endoscopy.

There are still conflicting data regarding the optimal bowel preparation for colonoscopy. This study was carried out to compare the tolerability, safety, and efficacy of three (two) widely used bowel lavage solutions in a large cohort of outpatients. Tolerability and safety were evaluated by means of a symptom questionnaire completed by each patient before the procedure. The scoring of cleanliness was performed during each examination by the blinded endoscopist. The treatment groups were comparable with regard to the baseline characteristics.

## Figures and Tables

**Table 1:** Cleanliness quality score of the blinded endoscopist.

0	Dry colon, no solid faeces
1	Only transparent fluid
2	Fluid faeces
3	Small amount of solid faeces, ≥90% of the mucosa visible
4	Solid faeces, <90% of the mucosa visible

**Table 2:** Characteristics of the patients separated by the cleansing protocol (A, B, and C).

	A	B	C	Total
Number	128	133	94	355
Age (median)	61	59	57.5	59
BMI (kg/m^2^)	25.1	24.9	24.9	25
Indication for colonoscopy
Abdominal symptoms	38.3%	40.6%	38.3%	139 (39.2%)
Other	7.8%	9.8%	7.4%	30 (8.5%)
Suspected colonic disorder	23.4%	24.1%	20.2%	90 (25.4%)
Control of colonic disease	32%	24.1%	20.2%	92 (25.9%)
Aggravation of colonic disease	5.5%	8.3%	8.5%	26 (7.3%)
Comorbidities	57.8%	49.6%	57.4%	194 (54.6%)
